# What Happens before Syncope? Study of the Time Frame Preceding Vasovagal Syncope

**DOI:** 10.5402/2011/659787

**Published:** 2011-04-14

**Authors:** Alfonso Lagi, Simone Cencetti, Alessandro Cartei

**Affiliations:** Dipartimento di Medicina Interna, Ospedale Santa Maria Nuova, Piazza S. Maria Nuova, 50100 Firenze, Italy

## Abstract

*Objective*. The events characterizing the very last part of the vasovagal crisis has not been determined. The aim of the study was to analyze the variations in respiratory pattern preceding the vaso-vagal syncope full-blown and the relationship between cardiovascular functions in order to assess the temporal sequence. *Methods*. Eleven consecutive patients were studied. Heart rate, arterial pressure, respiratory frequency, tidal volume, carbon dioxide, and oxygen saturation in time domain from supine and standing recordings were analyzed. *Results*. The respiratory activity is different in the time frame preceding syncope, both in V_T_ and breathing rate, and that the increase of the lung ventilation does not influence the baroreflex control during the presyncopal period but may be cause of the baroreflex failure during the full-blown syncope.

## 1. Introduction

The sequence of the events characterizing the last part of the vaso-vagal crisis preceding the full-blown vaso-vagal syncope (VVS), that is, hypotension, bradycardia, and loss of consciousness, has not been determined. Previous studies underlined that the full expression of the vaso-vagal reaction is preceded by changes in the respiratory pattern, that is, hyperventilation, hypocapnia, and cerebral vasoconstriction, in the last three minutes preceding bradycardia and hypotension [1–6]. Nonetheless, the respiratory pattern, the hierarchy existing between the changes in respiratory and cardiovascular signals before VVS, and their reciprocal influence need to be addressed.

Reciprocal and complex influences exist between cardiovascular and respiratory activity [7–11]. Low and high threshold lung mechanoreceptors have different and opposite effects on sympathetic activity in the cardiovascular system. On the other hand, chemoreflex influences both lung activity and autonomic system. Therefore they must be considered together in order to understand the pathophysiologic changes occurring during neuromediated syncope.

We studied baroreflex control by assessing relationship between RR interval and systolic arterial pressure (SAP) before syncope and whether the changes in respiratory frequency (RF) and tidal volume (V_T_) preceded the cardiovascular signals and also influenced them.

Therefore the aim of the present study was to analyze the respiratory variations preceding the full-blown VVS and the relationship between the cardiovascular functions, heart rate (HR) and SAP, in order to determine their temporal sequence.

## 2. Methods

### 2.1. Patients

Eleven consecutive patients (7 females) of mean age 29 years (range 21–32 y.o.) were studied. Each subject suffered from frequent VVS in the last year (more than three episodes) and in the preceding four years (more than two episodes per year). None of them was taking any drug, neither had smoking habits, and they were free from any other known disease. They were tested late in the morning, fasting from the evening before.

### 2.2. Procedure

Each patient was placed supine on a tilt table (Electro-Werk, type 1.73-0006, Hanning, Germany) in a quiet room and trained two times from supine to standing position, in order to get acquainted with the procedure and to reduce the emotional component. Room temperature was kept constant at 20–22°C, and the subjects were breathing in room air. Baseline recordings (3 minutes) were performed in the supine position. Afterwards the tilt table was turned up and the recording was started at 80° and maintained for 45 minutes or prematurely interrupted if hypotension and bradycardia occurred.

### 2.3. Data Acquisition and Analysis

The following signals were recorded directly and continuously on a personal computer (Macintosh IIx, Apple, Coupertino, USA) by a 12 bit analogue to digital converter (NBMio-16 board, National Instruments, Austin, Tex, USA; sampling rate 600 samples/s/channel): ECG (lead II), respiratory signal frequency (by an inductive thoracic belt), V_T_ (Master Pneumotachograph Oxicon Alpha-Jager Erich), respiratory gas (CO_2_ end Tidal was measured at the mouth by Novametrics Medical Systems, Wallingford, CT; O_2_ Saturation percentage by a transcutaneous device), and noninvasive arterial blood pressure (Finapres, Ohmeda 2300, Englewood, USA). Data were stored and analyzed with a dedicated software.

Data obtained in supine position (*Supine*) were analyzed separately from those in standing.

Heart Rate, SAP, V_T_, and respiratory frequency were calculated. Tidal volume (V_T_) was obtained breath by breath through integration of the pneumotachographic signal and was expressed as percentage of baseline value. An automatic interactive program allowed to identify each breath and to calculate the corresponding respiratory frequency: this was expressed as a mean of the breaths of the period, standard deviation, and range.

Previous studies confirmed that the 180 seconds before the onset of syncope were characterized by a significantly faster respiratory rate [2, 3] as compared to the beginning of tilt and subsequent periods. The standing recording was therefore divided in two segments of 100 seconds each, the first one representing the early orthostatic phase, indicated as orthostatic period, that is, 280–160 seconds before syncope and the second one representing the late orthostatic phase, which precedes the syncope, indicated as Presyncopal Period, that is, 100 seconds before syncope. A baseline supine recording of 100 seconds duration was analyzed.

The parameters in time and frequency domain were calculated in the three recorded segments.

Results were expressed comparing the Supine position with the orthostatic period and between this last and the presyncopal period.

### 2.4. Time Domain Analysis

Mean values and total variability were calculated for each one of the studied variables (RR interval, SAP, V_T_, and respiratory frequency) in the three periods. Respiratory gas was recorded breath by breath.

Furthermore each one of the three periods was split in five time frames of 20 seconds each, and the mean values were then compared. This analysis allowed to follow the time course of the events in each variable measured (RR, SAP, respiratory frequency and V_T_, CO_2_ and O_2_ Sat%).

### 2.5. Statistics

Comparisons of the three analyzed phases were made by means of two-tail paired *t*-test. Significance was set for *P*-value <  .05.

## 3. Results

### 3.1. Time Domain

**Figure 1:**
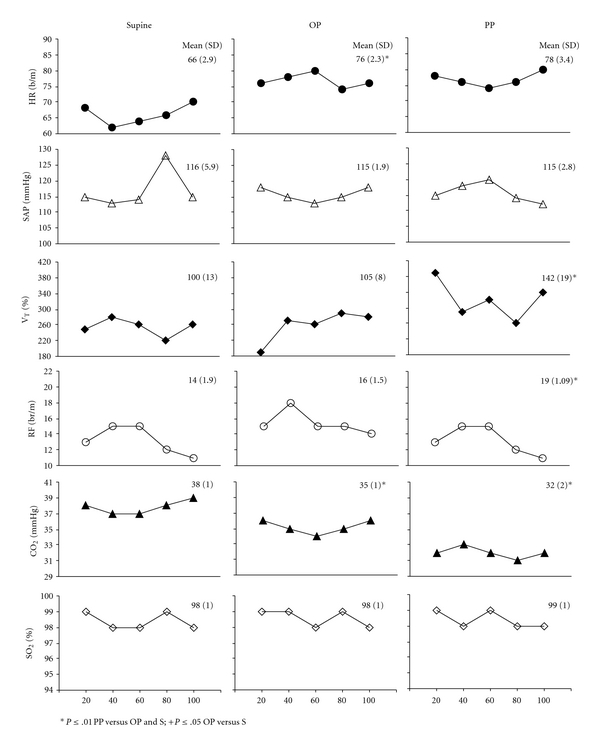
Mean of heart rate, systolic arterial pressure, tidal volume, respiratory frequency, CO_2_ and O_2_ saturation in different study periods. Abbreviations: VVS: vaso-vagal syncope; SAP: systolic arterial pressure; RF: respiratory frequency; V_T_: tidal volume; HR: heart rate; OP: early orthostatic phase; PP: preceding phase.

The changes of cardiovascular parameters, respiratory frequency and V_T_ are represented in Figure 1 as mean of beat by beat resumed in segment of 20”:

- Heart rate significantly increased from supine to orthostatic period (from 66 to 76 bpm) and did not change from orthostatic period to presyncopal period (from 76 to 78 bpm).
- SAP had not significant differences in supine versus orthostatic period and presyncopal period.
- V_T_ had significant difference in presyncopal period versus orthostatic period and supine. The mean value of V_T_ was 142% versus 105% and 100%, respectively (Figure 1).
- Respiratory frequency increased in presyncopal period versus supine and orthostatic period (19 versus 13 and 16 breaths per minutes, resp.).
- pCO_2_ significantly decreased in orthostatic period versus supine (35 versus 38 mmHg) and in presyncopal period versus orthostatic period (32 versus 35 mmHg).
- O_2_ saturation did not change.

## 4. Discussion

The main feature of the study was the characterization of the different patterns in cardiovascular and respiratory signals in the period preceding the full-blown VVS. Significant differences for V_T_ (Figure 1), for respiratory frequency and pCO_2_ (Figure 1) allowed to distinguish the presyncopal period versus the orthostatic period and supine. significant differences were found for Heart Rate (Figure 1) in the orthostatic period versus supine.

We studied arterial blood pressure, heart rate and respiratory frequency in order to investigate two events: the first one concerning the relationship between the respiratory activity, that is, respiratory frequency and V_T_, and the other cardiovascular parameters [12]. The study was carried out by head up tilt table test, the subjects were not taking any drugs in order to avoid pharmacological interferences, and the study population was selected for recurrent syncopal episodes. The study design was chosen in such a way that each patient could be compared with himself in different periods with the purpose of identifing the differences of studied variables. Time analysis was comparable among the three chosen periods and the parameters were constant in each period: in Presyncopal period the recording was interrupted before the onset of hypotension.

### 4.1. Analysis of Respiration

Changing position from supine to standing significantly modify respiratory activity. Respiratory frequency (from 14 to 16 breaths per minute), and V_T_ were increased (Figure 1) while pCO2 (from 38 to 35 mmHg) decreased. 

Respiratory activity shows a further change in presyncopal period, represented by the increase of respiratory frequency (from 16 to 19 breaths per minute, Figure 1) and V_T_ (42% increase from the baseline value) and by the decrease of pCO_2_ (from 35 to 31 mmHg) (Figure 1).

These overall results seem to suggest the following conclusions:

- there is a change in respiratory activity during the three different periods of the study;
- the increase of lung ventilation, expressed by the values of respiratory frequency and V_T_, characterizes the last period before syncope (i.e., Presyncopal Period);
- pCO_2_ is reduced as an effect of increased lung ventilation.

Normally respiration is coupled to heart rate and this phenomenon causes the respiratory sinus arrhythmia. The increase in V_T_ would modify the cardiovascular parameters by increasing respiratory arrhythmia [13, 14]. This finding demonstrates that the respiration pattern in presyncopal period, characterized by increasing of respiratory frequency and V_T_, has no influence on RR rhythm and SAP.

### 4.2. Baroreflex Function

The presence of hypotension and bradycardia in the full-blown phase of syncope is index of baroreflex failure meaning the failure to support the normal cardiovascular function.

The baroreflex function, explored by the *α* index, has normal and physiological pattern in all phases preceding the full blow of syncope. This is unmodified in presence of increased respiratory frequency and V_T_, in other words their increase is not able to modify baroreflex function in any periods, particularly in Presyncopal Period. Hyperventilation in Presyncopal Period, whose objective is the increase of blood venous return to heart to maintain an adequate SAP and cerebral perfusion [15], precedes the blunted baroreflex gain.

### 4.3. Is Hyperventilation a Compensator Factor in the Phase Preceding Syncope?

The physiological significance of the respiratory activity before the full blow of VVS and the best way to typify remains to be understood.

It is possible that the increase of V_T_ had a finalistic function, that is, increasing the venous blood afflux to thorax during the preparatory phase in which the pooling of blood in the subdiaphragmatic vessels network reaches its maximum level. The dissociation between respiratory activity and cardiovascular signals may be explained as the effect of the increase of V_T_ on different control systems such as pulmonary receptors [12], sympathetic activity, or cerebral vascular resistance [3].

**Figure 2:**
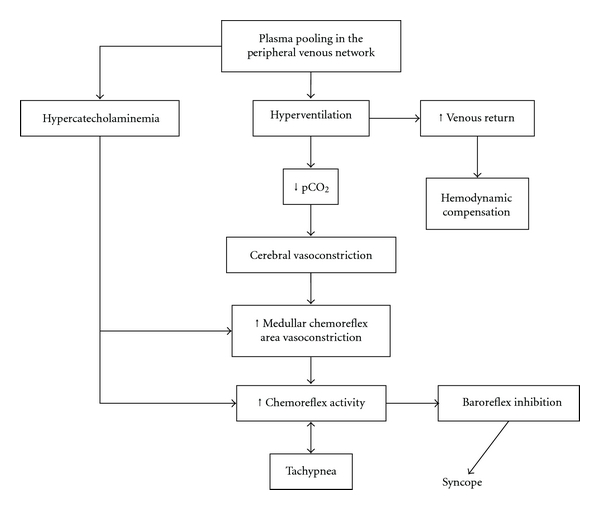
Hypothetical steps of baroreflex failure in syncope.

A conclusive hypothesis, shown in Figure 2, is that the venous return is able to stimulate the low pressure baroreceptors found in atria and in the pulmonary vessels. Sympathetic activity and increase of ventilation are the main components sustaining the venous return by the effect of the thoracic pump [13]. The higher V_T_, the greater the increase of the venous return, meaning that an inverse correlation between breathing rate and pressure fall exists. This may suggest that the increase in ventilation is more efficient in maintaining blood pressure if the breathing rate is slower [14, 16]. The importance of breathing in the time frame preceding syncope has been highlighted [2, 3]: the appearance of high respiratory activity is an index of inefficient compensatory action and comes before syncope.

During presyncopal period, the increased Respiratory Frequency associated to the increase of pulmonary ventilation, triggers the subsequent events represented by hyperventilation-induced hypocapnia leading to cerebral vasoconstriction [3] also in the chemoreflex areas [2]. This respiratory pattern is an index of increased chemoreflex gain that, through reciprocal influences, [9–11, 17] leads to baroreflex failure in the full expression of VVS. Presumably, in Presyncopal Period, the stimulation of high-threshold lung stretch receptors takes place, thus causing the following events (i.e., hypotension, bradycardia, and apnea), through their capability to inhibit sympathetic efferent activity [12, 18]. It remains to define the reciprocal influence between chemoreflex activity and baroreflex failure.

## 5. Conclusion

Data demonstrate that between the two orthostatic periods, particularly in the one preceding syncope, baroreflex function does not play an important role in the development of syncope because heart rate and SAP do not seem to be able to influence each other. This affirmation is not original because other observations came to this conclusion even though any case study has not been carried out yet [19].

The novel view these data achieve is that the respiratory activity is different in the time frame preceding syncope, both in V_T_ and breathing rate. The increase of the respiratory activity not related to heart rate and systolic pressure, sign of chemoreflex hyperfunction, may be cause of the baroreflex failure during the full blow of syncope. Indeed, the baroreflex function is ruled out from chemoreflex hyperfunction and not from mutual influence of SAP and heart rate.
